# Immunomodulatory Role of Vitamin D on Gut Microbiome in Children

**DOI:** 10.3390/biomedicines11051441

**Published:** 2023-05-14

**Authors:** Anika Tabassum, Adli Ali, Farah Dayana Zahedi, Noor Akmal Shareela Ismail

**Affiliations:** 1 Department of Biochemistry, Faculty of Medicine, Universiti Kebangsaan Malaysia, Kuala Lumpur 56000, Malaysia; p124173@siswa.ukm.edu.my; 2 Department of Pediatric, Faculty of Medicine, Universiti Kebangsaan Malaysia, Kuala Lumpur 56000, Malaysia; adli.ali@ppukm.ukm.edu.my; 3 Department of Otorhinolaryngology, Faculty of Medicine, Universiti Kebangsaan Malaysia, Kuala Lumpur 56000, Malaysia; farahdayana@ukm.edu.my

**Keywords:** Vitamin D, gut microbiome, immune system, taxa, children

## Abstract

Vitamin D plays a role in regulating the immune system and can be linked to the alteration of the gut microbiome, which leads to several immunological diseases. This systematic review aims to explore the relationship between Vitamin D and children’s gut microbiome, as well as its impact towards the immune system. We have systematically collated relevant studies from different databases concerning changes in the gut microbiome of children from infants to 18 years old associated with Vitamin D and the immunological pathways. The studies utilized 16S rRNA sequencing analysis of fecal matter with or without Vitamin D supplementation and Vitamin D levels. Ten studies were selected for the review, among which eight studies showed significant alterations in the gut microbiome related to Vitamin D supplementation or Vitamin D levels. The taxa of the phylum Actinobacteria, Bacteroidetes, Firmicutes, and Proteobacteria are the most altered in these studies. The alteration of the taxa alters the Th1 and Th2 pathways and changes the immune response. We will discuss how Vitamin D may contribute to the activation of immune pathways via its effects on intestinal barrier function, microbiome composition, and/or direct effects on immune responses. In conclusion, the studies examined in this review have provided evidence that Vitamin D levels may have an impact on the composition of children’s gut microbiomes.

## 1. Introduction

The human gut is a reservoir to a rich and diverse community of microbes [1]. The population of microorganisms inhabiting the gut is known as the gut microbiota, and the genome of those microbes refers to the gut microbiome [2]. The gut microbiota plays a vital part in our well-being and illnesses by metabolizing energy [3], producing short chain fatty acids (SCFA) [4] and vitamins [5] while regulating the immune system [6]. The genomic composition and diversity of the human gut is also responsible for childhood development, which includes the development of the immune system and related diseases [7].

The development of the microflora begins at birth, which is passed down from the mother and continues to develop throughout life but becomes comparatively stable when one is older [8]. The infant microbiome is the most susceptible to change, which makes prenatal and the first few years of microbiome development the most vital. The initial microbiota can regulate the infant epithelial gene expression, which determines the adult microbiome balance at a later age. Thus, reviewing the microbiome at an earlier age could control the immune system, as it is the initial stimulus at infancy. The childhood microbiome has various antigens, such as lipoproteins and polysaccharides, which help establish the immune system. Different compositions of microbes will form the immune system differently and it will also affect the immune system related diseases in that individual in childhood and in later life [9].

Immunological diseases have been associated with dysbiosis in children, in which the microbiome composition has been altered [10,11]. Diseases, such as Inflammatory Bowel Diseases (IBD) [12], allergic diseases [10], pediatric multiple sclerosis [13], Type 1 diabetes (T1D) [11], and asthma [14], are commonly associated with dysbiosis in children. Thus, the development of a healthy pediatric gut microbiome is important to prevent diseases and maintain better health. Many factors, such as children’s diets, antibiotic exposure, breast milk, or formula feeding of infants, can shape the gut microbiota composition and their function [15,16,17,18]. One of the most discussed factors affecting the gut microbiota has been the role of Vitamin D [19]. In past years, metagenomics has enabled researchers to take a comprehensive look into the human gut microbiome, which allows us to observe the shift in the composition of microbiomes associated with Vitamin D supplementation [20]. These analyses help us to elucidate the composition and diversity of the microbes, including their evenness, richness, and functional capacity. Several studies included high throughput 16S rRNA sequencing of the stool sample to successfully analyze the composition and detailed functional gene expression profile of the gut microbiome [21,22]. This 16S rRNA technique uses 16S rRNA as a marker to sequence genes, which has conserved hypervariable regions that can specifically identify the bacteria [23]. Studying the gut microbiome profile during childhood plays an important role in identifying the abundant bacteria and determining the effect of dysbiosis in children’s health and immunological diseases in relation to Vitamin D [24].

Vitamin D levels have been associated with improved health and a reduced incidence of immunological diseases in pregnancy and children [25]. Supplementation in deficient children with Vitamin D reduced diseases like asthma, allergic rhinitis, and IBD [26,27,28]. Additionally, Vitamin D has been suggested to be a key player in altering the composition of the gut microbiome. The immunological and anti-inflammatory role of Vitamin D could lead to the regulation of microbiomes. Studies have shown that Vitamin D regulates several expressions of immune cells [29], including immunoglobulin E (IgE), T-helper 1 (Th-1), and T-helper 2 (Th-2) pro-inflammtory and anti-inflammatory cytokines [30,31], and further maintains the role of the gastrointestinal barrier, which can alter the microbiome [32,33].

The characterization and function of the children’s gut microbiome are varied and must be observed, especially concerning the shift in the microbiota with their Vitamin D levels and supplementation among healthy children. There have been limited studies focusing on children’s gut microbiome associated with Vitamin D supplementation. Therefore, to investigate the immunomodulatory role of Vitamin D on the gut microbiome, we reviewed clinical and observational studies. This systematic review specifically focused on the prenatal and children’s Vitamin D levels or supplementation impact on the gut microbiome of the pediatric population.

## 2. Materials and Methods

### 2.1. Study Protocol

We followed the Preferred Reporting Items for Systematic reviews and Meta-Analyses (PRISMA) guideline for systematic reviews to conduct this review. Database search was conducted to find articles from January 1996 to January 2023 from the following databases: (1) PubMed, (2) Medline, (3) Scopus, and (4) Web of Science. Studies were also obtained by looking through reference lists in Google Scholar. Limits were applied during the search, including full-length text articles in English. The terms used for the search were: “Vitamin D and microbiome”, “Vitamin D and microbiome and immune”, “Vitamin D and microbiome and infection”, “Vitamin D and microbiome and inflammation”, “Vitamin D and microbiome and profiling”, “Vitamin D and microbiome and transcript”, and “Vitamin D and microbiome and pediatric”, “Vitamin D and microbiome and children”. There was no further retrieval of articles needed. No studies were excluded, and no additional analysis was conducted in order to reduce the risk of biasness. To keep the heterogeneity low, the methodology of the studies was kept constant. The detailed search strategy is provided in Appendix A.

### 2.2. Eligibility Criteria

The abstracts of each article were reviewed initially to check for eligibility. The studies that were included were clinical trials, randomized controlled clinical trials, and cohort studies. The children included were not limited to healthy children. Studies including children ranging from infants to 18 years of age were selected. The selected studies contained a measure of Vitamin D levels, supplementation of Vitamin D, or prenatal supplementation of Vitamin D. The outcome of the studies was recorded as the measure of variability within a sample (alpha diversity) and between samples in microbial composition (beta diversity), species richness, prevalence, and/or (relative) abundance of bacterial taxa. The studies were excluded if they did not analyze the gut microbiome with 16S rRNA gene sequencing. The studies and reviews that were in any language other than English were also excluded.

## 3. Results

### 3.1. Study Selection

**Figure 1:**
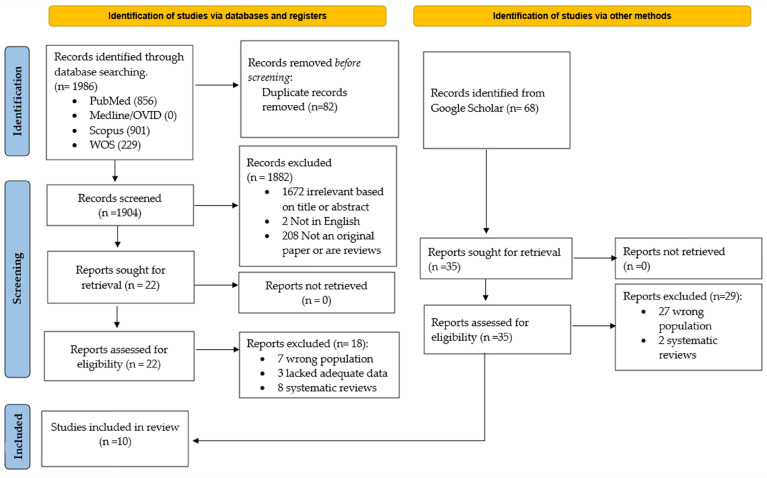
Flowchart to show study selection procedure.

Across all databases, 2072 articles were obtained, and 82 duplicates were removed. The titles were then skimmed to select 22 articles from PubMed, Medline, Scopus, and WOS, as well as 35 articles from Google Scholar, according to their relevance. Next, the abstract and full articles were studied to exclude the articles that did not fall into the eligibility criteria. Two reviewers (AT, NASI) independently searched for the articles. Twenty-two full-text articles were screened from different databases, and 35 full-text articles were screened from Google Scholar. We selected 10 studies for this review. A flowchart for article identification, screening, and final selection is given below (Figure 1).

### 3.2. Study Characteristics

Out of the 10 studies, eight studies included administering Vitamin D supplementation, and two studies included looking into the blood Vitamin D levels. Two studies included administering prenatal Vitamin D supplementation of 400 IU versus 4000 IU [34] and 400 IU versus 2800 IU [35]. One study included administering Vitamin D supplementation of 400 IU or placebo to infants [36]. One study did not include the amount of Vitamin D supplementation to infants [37], while two studies included administering Vitamin D supplementation to both mothers and infants. In one of these two, mothers received a dose of 0 IU versus < 400 IU/day versus ≥ 400 IU/day, while the dosage for infants was not mentioned [38]. In the other, mothers and infants were both were given 400 IU/day. Overall, one study included supplementing high doses of Vitamin D to adolescent girls 50,000 IU/week [39]. One study included providing Vitamin D 400 IU/day or Vitamin D 400 IU/day with Vitamin D 200 or 400 IU/day with another dosage of Vitamin D 400 IU/day or 400 IU/day with 800 IU/day [40]. One out of the 10 studies included Vitamin D levels in 4-to-14-year-old children [41]. One study had maternal and infant vitamin D supplementation of >400 IU/day or <400 IU/day or 400 IU/day [42]. Another study examined the maternal and prenatal Vitamin D levels [43]. The studies are included, and their characteristics are summarized in the table below (Table 1). The primary outcome of the investigations was documented as an abundance of bacterial taxa and alpha or beta diversity of the samples. There were no requirements for any data conversion. In all studies, the gut microbiome measurement was conducted using fecal samples. All of these studies utilized 16S rRNA gene sequencing from fecal samples to reduce the variability in the microbiome measurement.

### 3.3. Association of Vitamin D with Microbial Composition and Diversity

Nine out of 10 studies showed a significant association between Vitamin D and the gut microbiome (Table 2). There was some evidence that Vitamin D was associated with bacterial community composition. Four studies [36,37,40,43] determined that supplementation with Vitamin D_3_ was associated with a shift in microbial composition. Two of these studies containing exclusively breast-fed infants showed a relation between Vitamin D supplementation and alpha and beta diversity. Vitamin D increased the microbial diversity and richness and showed a significant beta diversity [36,37,40,43]. On the contrary, a study in infants with eczema showed a significant low microbial diversity with high doses of Vitamin D but had similarities between the low-Vitamin D-supplemented group and healthy infants [40]. A positive association with the infant gut microbial community with Vitamin D status was observed in some of these studies.

### 3.4. Vitamin D Effects on the Phyla

Actinobacteria, Bacteroidetes, Firmicutes, and Proteobacteria were recorded as the most prevalent phyla in some of the studies [34,37]. All the studies showed alterations in the taxa of these phyla: Actinobacteria (Bifidobacterium spp and Corynebacterium), Bacteroidetes (Prevotellaceae, Prevotella, Prevotella copri, Alistipes, *Bacteroides* spp., Bacteroides massillensis, Bacteroides caceae, Bacteroides eggerthii, Bacteroides plebeius, Bacteroides vulgatus, and B. fragilis), Firmicutes (Lachnospiraceae, unclassified clostardilas, Lactococcus, Lactobacillus, Ruminococcus gnavus, C.difficile, Enterococcus, Acidaminococcales, Phascolarctobacterium, Phascolarctobacterium faecium, and Megamonas), Proteobacteria (Haemophilus, Bilophila, Sutterellaceae, Parasutterella, Parasutterella excrementihominis, and Acinetobacter) (Table 3).

There was a co-abundance of Firmicutes, Proteobacteria, Bacteriodetes, and Veillonella in children aged 3 to 6 months belonging to a parent with allergy or asthma [34]. Vitamin D levels increased the co-abundance of Firmicutes (Lachnospiraceae/Clostridiales) in these children and decreased Bacteroides. Firmicutes were significantly lower in Vitamin D-deficient children [42] but increased after supplementation in adolescent girls [40]. For breast-fed infants with 4 months supplementation of Vitamin D, they showed a significant increase in the abundance of Actinobacteria and a decrease in Bacteroidetes [37] in comparison with breast-fed infants without Vitamin D supplementation. Bacteroidetes and Lactobacillus were reduced by 72% and 24%, respectively, and Firmicutes and Enterococcus were increased [40] after high doses of Vitamin D supplementation. Proteobacteria was found to be the most abundant gut microbiome at birth. Infants whose mothers were supplemented with Vitamin D and breast-fed infants had decreased amounts of Proteobacteria, which is linked with obesity [43]. Children who were deficient in Vitamin D levels showed an elevated Bacteroidetes to-Firmicutes (B/F) ratio, which regulates gut microbiota homeostasis and also lowered the Bacteroides-to-Prevotella ratio, which is the main enterotype of the gut microbiome [41].

### 3.5. Alteration of the Other Taxa with Vitamin D

In the level of genus, Bifidobacterium was seen to be the most dominant genus present in faecal samples in three studies, regardless of any association with Vitamin D [36,37,40]. Bifidobacterium increased significantly after high doses of Vitamin D supplementation in 12-to-18-year-old females [40], and it inversely correlated with maternal multivitamin supplementation containing Vitamin D [39] and was higher in Vitamin D-supplemented breast-fed children.

Bacteroidetes and its species Bacteroides massillensis were increased in Vitamin D-deficient children [42], and some of its species (Bacteroides caceae, Bacteroides eggerthii, and Bacteroides plebeius) were lower in Vitamin D-deficient young children. Bacteroides were also lower in Vitamin D-supplemented infants. Prevotella was elevated in Vitamin D-deficient children, including species Prevotella dislens and Prevotella bivia [42]. Prvotella, its family Prevotellaceae, and species Prevotella copri were lowered, and the family Sutterellaceae, genus Parasutterella, and species Parasutterella excrementihominis increased with Vitamin D supplementation in children with eczema [40].

The genus Bilophila was found to be lowered in infants with prenatal Vitamin D supplementation [43]. *Alistipes* was determined to be significantly reduced in children with Vitamin D deficiency, including some species of the genus like, *Alistipes finegoldii*, *Alistipes* sp. AL1, and *Alistipes* sp. N15.MGS–157 [41], whereas some of the species of the genus increased in children with Vitamin D deficiency, for example, *Alistipes* marseille and *Alistipes* indistinctus [40]. Vitamin D supplementation had a significant reduction of Lactobacillus in adolescent girls [40] and increases in infants who were supplemented with Vitamin D [37]. Enterococcus Increased [40] and Streptococcus were found elevated with Vitamin D supplementation [37].

Maternal consumption of Vitamin D-fortified milk reduced the likelihood of C. difficile colonization in infants. C. difficile inversely correlated with prenatal multivitamin supplementation containing Vitamin D, and it was reduced in Vitamin D-supplemented infants who were breast-fed [39]. Prenatal maternal Vitamin D supplementation was not associated with infant *C.* difficile colonization in crude or adjusted models [43]. Order Acidaminococcales, genus Phascolarctobacterium, and species Phascolarctobacterium faecium increased with Vitamin D supplementation in infants with eczema [40].

Corynebacterium was higher with Vitamin D levels. Haemophilus was higher in prenatal supplementation and was found lower in infants who were supplemented with Vitamin D. Cord Blood Vitamin D levels were related with high Lachnobacterium but low Lactococcus [41]. Megamonas decreased in Vitamin D supplementation in infants [42], while Ruminococcus gnavus decreased with Vitamin D levels at the time of delivery and increased with prenatal Vitamin D levels [43].

## 4. Discussion

### 4.1. Pediatric Gut Microbiome and Immune System

In this review, we intended to study selected articles with changes in the gut microbiome in children with Vitamin D supplementation and different Vitamin D levels. The studies showed an association between Vitamin D and the intestinal microbiota in children, while the phyla Firmicutes, Bacteroidetes, Actinobacteria, and Proteobacteria were observed to be altered with Vitamin D. In diseases like NAFLD and ulcerative colitis, these phyla are considered important in the diseases’ development [44,45]. A healthy pediatric gut microbiome is said to be dominated by Firmicutes, Bacteroidetes, Actinobacteria, and Proteobacteria [46,47]. An alteration of the taxa of these phyla are related with dysbiosis [48], which is related to diseases. A healthy gut microbiome has commensal and limited pathogenic microbes. The gut barrier plays an important part in maintaining a healthy microbiome by restricting pathogen entry. The immune cells can differentiate between commensal and pathogenic microbes with the help of the intestinal mucosal barrier, which reduces pathogenic reproduction [49]. Furthermore, the impermeability of the intestinal barrier helps reduce the adherence of the pathogens. The commensal bacteria, which have a neutral relationship with the host, can produce microbial metabolites such as bile acids, lactate, lipids, amino acids, vitamins like the Vitamin B complex, and SCFAs [3,50]. These gut microbiome metabolites are the major mediators for the immune system. For example, Parasutterella produces aromatic amino acid metabolites, which enhance gut barrier integrity [51]. SCFAs are also significant in keeping the integrity of the intestinal epithelial layer intact by stabilizing hypoxia-inducible factor 1, HIF-1. Higher partibility of the intestinal epithelium results from lower SCFAs, such as butyrate [52], restricting pathogen entry [49]. Thus, SCFAs and aromatic amino acids can help reduce dysbiosis and maintain intestinal homeostasis. This is supported by one of the selected studies where we saw SCFA-producing bacteria, acidaminococcus, and bacteroides vulgatus, while amino acid-producing bacteria Parasutterella were reduced in infants with eczema compared to healthy infants. The abundance of acidaminococcus, bacteroides vulgatus, and Parasutterella was elevated after Vitamin D supplementation in the infants in this study [40].

Overall, this implies that Vitamin D has an impact on the abundance of commensal bacteria. Commensal bacteria abundance is important to prevent pathogens, as these bacteria can compete with pathogenic bacteria for nutrients. Plus, Vitamin D receptors are present in the gut barrier and induce the production of antimicrobial peptides (AMPs) [53]. The Paneth cells in the GI tract release AMPs which kill pathogens by destroying their cell membranes. For instance, AMP α-defensins consist of human defensin 6, which does not allow the pathogenic bacteria to invade the gut, whereas β-defensins disrupt their cell membrane, and cathelicidin inhibit pathogen colonization [54]. AMPs modulate pH in order to prevent pathogens and reduce infectious diseases. Therefore, Vitamin D can alter the composition of the gut microbiome and the regulation of AMPs by Vitamin D, which leads them to modulate the immune system and reduce infections [55].

Vitamin D can also regulate the inflammatory response via SCFA production. Bacteria that produce SCFAs have an anti-inflammatory effect, which reduces inflammation. Butyrate raises transcription factor forkhead box P 3 (Foxp3) expression by T regulatory cells and histone acetylation [54], contributing further to the anti-inflammatory response. Additionally, it reduces nuclear factor-kappaB (NF-kB) [56], all of which reduces the production of pro-inflammatory cytokines. The increase of SCFA releasing bacteria by Vitamin D increases SCFA-influenced B-cell secretion of immunoglobin A (IgA). IgA plays an important part in maintaining gut barrier immune response as it reduces macrophages and dendritic cells and restricts chemotaxis in neutrophils [57]. Acetate-modulated IgA binds with specific microbes [58] as they take part in modulating inflammatory responses by elevating anti-inflammatory interleukin (IL), IL-10 and IL-18 as well as decreasing pro-inflammatory IL-1, TNF alpha, IL-6, IL-8, and IL-12 [57,59]. In the selected studies, Vitamin D reduced pro-inflammatory bacteria Bacteroidetes. Bacteroidetes and its genus Prevotella influence a pro-inflammatory response by releasing lipopolysaccharides, which activates macrophages and enhances inflammation. Prevotella was observed to be high in inflammatory disease, like eczema, and linked with declining health.

### 4.2. Vitamin D and Immune Response

Along with the modulation of the composition of the gut microbial flora, Vitamin D also regulates the human immune response towards microbiota. Vitamin D and its nuclear hormone receptor, VDR, can be expressed in the gut [34] and in immune cells like B and T lymphocytes [60]. The Vitamin D and VDR complex modulate the immune system by increasing some immune cells such as natural killer (NK), regulatory T cells (Treg), and IL-10, and decreasing some that include cells like neutrophil, IL-6, and IgE [61]. In one of the studies, the children with eczema had higher IL-6 and IgE levels, and after Vitamin D supplementation, both IgE and pro-inflammatory IL-6 were reduced to near-normal levels [40].

Vitamin D decreases the maturation of dendritic cells, increases macrophage M2, and reduces macrophage M1 phenotype. M1 activity inhibits cell proliferation and causes tissue damage, while M2 activity promotes cell proliferation and tissue repair. M1 macrophage releases TNF-a, IL-6, and cyclooxygenase-2 (COX2), which causes inflammation [62]. Vitamin D blocks the NF-kB pathway and COX2 transcription. This results in the reduction of reactive oxidative species. If not reduced, ROS can cause oxidative stress [63], DNA damage, and cell death.

The Vitamin D/VDR complex promotes T cell proliferation to form Treg cells and increase Th-2 pro-inflammatory cytokines, such as IL-10 cells. It also reduces Th-1 and Th-17 pro-inflammatory cytokines, such as IL-12 and IL-17. The increase of Th17 causes autoimmune diseases and allergies. As Vitamin D is responsible for increasing Treg cells and reducing cytokines IL-23 and IL-6, it reduces Th-17. It is also accompanied by the increase of Foxp3. Vitamin D decreases IFN-y, and that in turn reduces Th-1. When Vitamin D increases IL-4, it helps elevate Th2. Vitamin D also controls the B regulatory cells, decreases IgE release by B cells, and increases IL-10 [64].

### 4.3. The Role of Vitamin D in Pediatric Gut Microbiome and Diseases

As Vitamin D is linked with changing the gut microbiome and influencing the immune response via the gut microbiome, Vitamin D might also affect the immunological diseases relating to gut microbiome. Previously, studies correlated Vitamin D deficiency with increased autoimmune diseases. For example, *Megamonas* spp. increased the risk of childhood asthma, and decreased Lachnospira was found in asthmatic patients. These taxa were altered with increased Vitamin D levels. One of the studies showed a decrease of Megamonas, and another showed an increase of Lachnospira with Vitamin D supplementation [43]. In childhood asthma, an influx of pro-inflammatory IL-17, IL-3, and IL-4, as well as an increase of Th-17 cells and an imbalance of Th1/Th2 cells, are found responsible for the disease [65]. The pro-inflammatory cytokines and Th-17 decrease with elevated SCFA-releasing bacteria. Dysbiosis due to low levels of Vitamin D can cause less SCFA, which reduces dendritic cell maturation and, in turn, affects Th17. The reduction of Il-17 was also seen in allergic rhinitis patients with Vitamin D supplementation [65]. The reduction of IL-17, Il-4, and IgE were all linked with higher Vitamin D levels and an improvement of allergic symptoms. Allergic pediatric patients have elevated Bacteroides and Alistipes [66], both of which are linked with reduced SCFAs. In our selected studies, we found a decrease in Bacteroides and Alistipes with increasing Vitamin D. 

Vitamin D deficiency was seen in Chinese children with Type 1 diabetes [67], and dysbiosis related to low Vitamin D levels was found in these children. This might be because butyrate reduction alters the tight junctions of the colon and causes leaky gut, which has been linked with diabetes. Lowering the abundance of Firmicutes can cause this imbalance of butyrate. In our search, Firmicutes phylum was observed to be increased with Vitamin D supplementation. Previous studies showed some Firmicutes were reduced in Cystic Fibrosis in children [68], and high amounts of some Firmicutes were related with obesity in children and C-reactive protein, which is an inflammation marker. In one of the studies, it was seen that the increasing Firmicutes and Bacteroidetes ratio was related with decreasing BMI [69]. Firmicutes and Bacteroidetes are said to be the two most significant phylum in the gut microbiome, to an extent that F/B ratio is considered as a determining factor of gut microbiome dysbiosis. An increase in the ratio can lead to IBD, and a decrease in its ratio can lead to obesity [70]. Similarly, we observed in one of the studies that the F/B ratio was lower in Vitamin D-deficient children. Relative to Firmicutes, Bacteroidetes was higher in deficient children, making a lower F/B ratio, which is known to maintain the balance of the microbiome [71]. The gut microbiota of 4–14-year-old children were profusely dominated by Prevotella. A higher Prevotella-to-Bacteroides (P/B) ratio was found in Vitamin D-deficient children, which has been linked with obesity [72]. Prevotella and Bacteroides are the main enterotypes that are usually found and help in classification of the intestinal microbiome. Hence, it indicates that Vitamin D supplementation can shift the enterotypes of the gut.

**Figure 2:**
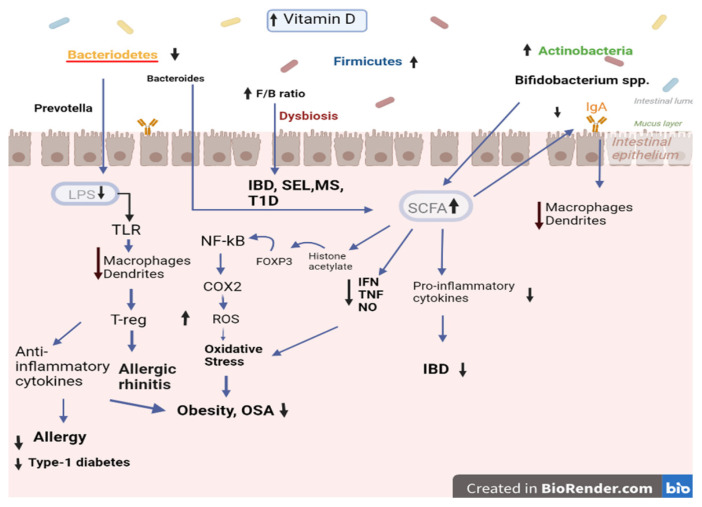
The role of Vitamin D in pediatric gut microbiome and diseases. Created with biorender.com (accessed on 13 January 2023).

High Vitamin D levels are linked with reducing inflammatory disorders in children, like Crohn’s disease [73]. A study suggests VDR can reduce inflammation in the ulcerative where hypoxia-inducible factor 1 is increased by blocking the NF-kB pathway [74]. Diseases like IBD, diarrhea, chronic inflammation, Type 1 diabetes, and infections are related to compromised gut epithelial layers [75]. The intestinal barrier integrity is upheld by the help of Vitamin D as it maintains which immune cells are being expressed in the tight junction, a layer that is significant in preventing bacterial invasion [76]. Basically, the Vitamin D/VDR complex suppresses apoptosis in the epithelium and restricts the inflammatory response to cause apoptosis in IBD patients. Vitamin D-influenced AMPs play critical roles in maintaining tolerance to gut microbiota as well, given that disruptions in tolerance to commensal microbiota and loss of barrier function play major roles in the pathogenesis of inflammatory bowel disease (IBD) [77]. The improvement of IBD symptoms after Vitamin D supplementation is consistent. NOD2, a nucleotide-binding oligomerization domain containing 2 from neutrophil-to-lymphocyte ratio (NLR) are linked with IBD. NOD2 can influence NF-kB as it binds with muramyl dipeptide and influences Transforming growth factor-β (TGF-β)-activated kinase 1 (TAK1). TAK1 is responsible for inititating NF-kB [78]. This results in autophagy. Vitamin D is associated with autophagy through the VD receptor (VDR). The loss of VDR impairs autophagy and enhances cell death through apoptosis. Dysbiosis in diseases like IBD is linked to NOD2 alleles, which is also related with Vitamin D [12,78]. Vitamin D can regulate AMP production. It has been demonstrated that the level of expression of AMP can influence the relative composition of the gut microbiome in α-defensins deficient mice. So, Vitamin D levels can change the gut microbiome (Figure 2).

### 4.4. Limitations

However, despite the evidence provided in this review that Vitamin D can influence the gut microbiome in children, there were some limitations to our study. Although we have done a thorough systematic search, we cannot exclude the possibility of not including papers outside the investigation protocol. Additionally, there was no statistical measurement performed. Even though there were not many methodological differences in the studies, this can be considered for further analysis.

## 5. Conclusions

The studies included in this review have shown evidence that the composition of the children’s intestinal microbiome could be altered by Vitamin D levels. These present data suggesting an indirect influence of Vitamin D on the children’s gut microbiome. The pediatric gut microbiome was altered in Vitamin D deficiency and supplementation during the prenatal and infancy period, as well as with older children. Therefore, Vitamin D plays an important role in altering the gut microbiome in children. Vitamin D supplementation has been determined to improve gut microbiome and the diversity in children, which can have a positive impact on their overall health and immune system function. Furthermore, when evaluating Vitamin D as a potential treatment for immunological illnesses in children associated with gut microbiota, such as allergies, asthma, and eczema, the evaluation of publications in this systematic review can be used as a source of information. No systematic review, to our knowledge, has reported and evaluated this link in children. However, more research is needed to fully understand the mechanisms behind the relationship between Vitamin D and gut microbiota and how this relationship can be utilized for therapeutic interventions. These data could potentially implicate future clinical studies to observe the direct effect of Vitamin D supplementation towards reducing the clinical symptoms of immunological diseases. Nonetheless, ensuring that children have adequate Vitamin D levels through diet or supplementation may be an important strategy for maintaining a healthy gut microbiome and promoting overall health in children.

## Figures and Tables

**Table 1:** Characteristics of the studies that use Vitamin D supplementation and its effect on the gut microbiome.

Title	Reference	Objective	Type of Study	Sample Size	Age Range	Lab Findings
Factors influencing the infant gut microbiome at age 3–6 months: findings from the ethnically diverse Vitamin D Antenatal Asthma Reduction Trial (VDAART).	[34]	To ascertain the effects of prenatal and early life factors on the gut microbiome in a sizable, ethnically diverse study population of infants who were enrolled in the Vitamin D Antenatal Asthma Reduction Trial, a clinical trial of Vitamin D supplementation in pregnancy to prevent allergies and asthma in offspring.	Cross-sectional (nested within an RCT)	333	3–6 months old	Lachnobacterium, Lachnospiraceae and U. clostridiales coabundance, and a decrease in Lactococcus, were all associated with Vitamin D levels in cord blood immediately following birth (in utero)
Prenatal dietary supplements influence the infant airway microbiota in a randomized factorial clinical trial	[35]	To determine if providing mothers with n-3 LCPUFA and Vitamin D influences the microbiome of the infant and mother and to clarify if the protective impact of these supplements on asthma can be regulated by the microbiota.	Random-ized clinical trials	580	Prenatal–1-year-old	No significant data
Metagenomic analysis of the gut microbiome composition associated with Vitamin D supplementation in Taiwanese infants	[36]	To ascertain the impact of Vitamin D supplementation on the development of the infant gut microbiome in Taiwan, as well as to look into the distribution and diversity of the infant gut microbiota under various feeding regimens, both with and without Vitamin D administration.	Clinical trials	62	0–4 months old	Infants that received Vitamin D supplements showed elevated levels of the genus Lactobacillus, Streptococcus, and Bifidobacterium, as well as a greater F/B ratio. Supplementing with Vitamin D decreased the amount of Bacteroides. The majority of the pathways identified were found to be engaged in various types of antibiotic production, which suggests that microbial communities exist in highly competitive environments, according to the metabolic analysis profiles in breastfed infants with Vitamin D deficiency.
Vitamin D Supplementation in Exclusively Breastfed Infants Is Associated with Alterations in the Fecal Microbiome	[37]	In order to ascertain the relationship between maternal and infant characteristics, infant feeding practises, and the gut microbiota profiles in 3- to 9-month-old infants, we separately analysed infants who were exclusively breastfed and those who were not in order to evaluate the impact of different feeding practises features on the microbiotas of the infants in each group.	Cohort study	191	3–9 months old	Infants who were exclusively breastfed and received Vitamin D supplements saw a decline in hemophilus
Influence of Vitamin D on key bacterial taxa in infant microbiota in the KOALA Birth Cohort Study	[38]	To determine if maternal Vitamin D administration, maternal plasma 25-hydroxyvitamin D concentration, or newborn Vitamin D administration directly affects major bacterial taxa within newborns’ microbiome at one month old.	Cohort study	913	1 month old	*Bifidobacterium* spp. decreased when maternal Vitamin D supplementation levels increased. C. difficile levels in breastfed infants with Vitamin D supplements were lower as well.
The effects of high doses of Vitamin D on the composition of the gut microbiome of adolescent girls	[39]	To assess the impact of a high-dose Vitamin D supplement on the gut microbiome’s composition.	Cohort study	50	12–18 years old	The expression folds of Enterococcus (1.05), Bifidobacterium (1.20), and Firmicutes (1.50) all increase with Vitamin D treatment. While Lactobacillus 24% (*p* = 0.006) and Bacteroidetes 72% (*p* < 0.0001) declined.
Effect of Different Doses of Vitamin D on the Intestinal Flora of Babies with Eczema: An Experimental Study	[40]	To determine whether altering the Vitamin D dosage can restore the immune system and intestinal flora of infants with eczema who typically consume 400 IU of Vitamin D per day.		32	1–12 months	With low Vitamin D dosage compared to no supplementation, Prevotella, species Prevotella copri, and family Prevotellaceae decreased in children with eczema. Children with eczema had higher levels of the bacteria Bacteroides vulgatus, Phascolarctobacterium faecium, order Acidaminococcales, family Acidaminococcaceae, genus Phascolarctobacterium, f-Sutterellaceae, and species Parasutterella excrementihominis with low Vitamin D supplementation compared to no supplementation.
Tipping the Balance: Vitamin D Inadequacy in Children Impacts the Major Gut Bacterial Phyla	[41]	To comprehend the incidence, causes of it, and effects of inadequate Vitamin D in Qatari children.	Cohort study	64	4–14 years old	Prevotella 9 and Bacteroidetes increased in Vitamin D-deficient children (50 nmol/L), but Firmicutes, Bacteroides, Alistipes, B/P ratio, and F/B reduced. Prevotella dislens, Prevotella bivia, Bacteroides massillensis, Alistipes marseille, and Alistipes indistinctus were among the species that were more prevalent. Bacteroides and Alistipes, including Alistipes finegoldii, Alistipes sp. AL1, Alistipes sp. N15 MGS-157, Bacteroides caceae, Bacteroides eggerthii, and Bacteroides plebeius, were more prevalent in Vitamin D non-deficient (>50 nmol/L) individuals.
Vitamin D supplementa-tion in pregnancy and early infancy in relation to gut microbiota composition and C. difficile colonization: Implications for viral respiratory infections	[42]	In 1157 mother–infant couples participating in the CHILD (Canadian Healthy Infant Longitudinal Development) Cohort Study from 2009 to 2012, the connection between maternal and newborn Vitamin D supplementation, infant gut microbiota makeup, and Clostridioides difficile colonisation was examined.	Cohort study	1157	3–4 months old	Genus Megamonas declines in infants receiving Vitamin D supplements. In breastfed newborns, Bilophila (-) and Lachnospiraceae decrease with maternal Vitamin D administration, but Haemophilus rises. Once breastfeeding status and other variables were taken into account, neither infant nor maternal Vitamin D supplementation was linked to C. difficile colonisation. Infant colonisation with C. difficile was less likely when mothers drank milk that was supplemented with Vitamin D.
Maternal and cord blood Vitamin D level, and the infant gut microbiota in a birth cohort study	[43]	To investigate relationships between newborn gut microbiota and plasma 25[OH]D levels throughout pregnancy or at birth (cord blood).	Cohort study	499	1–6 months old	Acinetobacter and Corynebacterium increased with maternal and cord Vitamin D. Ruminococcus gnavus decreased with Vitamin D treatment in Cord. Supplementing with prenatal Vitamin D led to a rise in Ruminococcus gnavus.

**Table 2:** Microbial diversity of the selected studies on Vitamin D levels in infants.

Reference	Dosage	Study Time	Placebo or Comparator	Health Condition	Alpha Diversity and Beta Diversity of the Gut Microbiota
[34]	4000 IU vitamin D + prenatal vitamins or 400 IU vitamin D + prenatal vitamins	18 months	Different doses	Infants of asthmatic or allergic parent	No significant differences in the alpha diversity or beta diversity of the infant gut microbiota (*p* > 0.05)
[35]	2800 IU/day or 400 IU/day + placebo	3 months	Different doses with Placebo	Wheezing or asthmatic mothers	No significant differences were observed between the Vitamin D supplementation group and the control group for the bacterial diversity at 1 week, 1 month, or 1 year postpartum (*p* = 0.955, *p* = 0.865, *p* = 0.971).
[36]	400 IU of vitamin D/day or placebo	4 months	Placebo	Healthy	For the beta diversity, the gut bacteria composition differed significantly at the different time points (ANOSIM, R = 0.742, *p* = 2.14 × 10^−5^). The alpha diversity index strongly reduced in infants during the period from birth to term age while having no obvious change after 1 month
[37]	Vitamin D supplement vs. no Vitamin D supplement in past 24 h	24 h	Vitamin D doses or no doses	Healthy	Alpha diversity: lower Shannon index (*p*-value < 0.007) than those infants who were not given a Vitamin D. Beta diversity: *p*-value = 0.02
[38]	Maternal VDS: 0 μg or <10 μg/day or ≥10 μg/day. Infant VDS: classified as yes or no	6 months	Different doses or no dose	Healthy	N/A
[39]	50,000 IU/week	9 weeks	Before and after supplementation	Healthy	N/A
[40]	Vitamin D 400 IU/day or vitamin D 400 IU/day + vitamin D 200 (D-LOW) or 400 IU/day + vitamin D 400 IU/day (D-MED) or 400 IU/day+ 800 IU/day (D-HIGH)	1 month	Comparison between doses	Healthy infants and infant with Eczema	For alpha diversity, no significant difference in the richness in the groups. Diversity in the D–HIGH group was lower (*p* < 0.01). For beta diversity, there were similarities between eczema and D–MID group, as well as control and D-LOW group. Other groups differed significantly.
[41]	N/A	N/A	Vitamin D deficient and non-deficient	Healthy	No significant difference in alpha diversity beta diversity measure (*p* < 0.01)
[42]	Maternal and infant VDS of >400 IU/day/<400 IU/day/400 IU/day	3 months	Different doses or no dose	Healthy	N/A
[43]	N/A	N/A	Vitamin D levels	Higher risk for asthma	Higher prenatal 25(OH)D level was significantly associated with decreased richness (*p* = 0.028) and diversity (*p* = 0.012) of the gut microbiota at 1-month infants. Prenatal and cord 25(OH)D levels were significantly associated with 1-month microbiota composition.

**Table 3:** Alteration of bacterial taxa by Vitamin D in the selected studies.

Phylum	Class	Order	Family	Genus	Species
Actinobacteria	Actinomycetia	Mycobacteriales	Corynebacteriaceae	Corynebacterium	
Actinobacteria	Actinomycetia	Bifidobacteriales	Bifidobacteriaceae	*Bifidobacterium* spp.	
Bacteroidetes	Bacteroidia	Bacteroidales	Prevotellaceae	Prevotella	Prevotella dislens
Bacteroidetes	Bacteroidia	Bacteroidales	Prevotellaceae	Prevotella	Prevotella bivia
Bacteroidetes	Bacteroidia	Bacteroidales	Prevotellaceae	Prevotella	Prevotella copri
Bacteroidetes	Bacteroidia	Bacteroidales	Rikenellaceae	Alistipes	Alistipes finegoldii
Bacteroidetes	Bacteroidia	Bacteroidales	Rikenellaceae	Alistipes	Alistipes sp. AL1
Bacteroidetes	Bacteroidia	Bacteroidales	Rikenellaceae	Alistipes	Alistipes sp. N15 MGS-157
Bacteroidetes	Bacteroidia	Bacteroidales	Rikenellaceae	Alistipes	Alistipes marseille
Bacteroidetes	Bacteroidia	Bacteroidales	Rikenellaceae	Alistipes	Alistipes indistinctus
Bacteroidetes	Bacteroidia	Bacteroidales	Bacteroidaceae	Bacteroides	Bacteroides massillensis
Bacteroidetes	Bacteroidia	Bacteroidales	Bacteroidaceae	Bacteroides	Bacteroides caceae
Bacteroidetes	Bacteroidia	Bacteroidales	Bacteroidaceae	Bacteroides	Bacteroides eggerthii
Bacteroidetes	Bacteroidia	Bacteroidales	Bacteroidaceae	Bacteroides	Bacteroides plebeius
Bacteroidetes	Bacteroidia	Bacteroidales	Bacteroidaceae	Bacteroides	Bacteroides vulgatus
Firmicutes	Clostridia	Eubacteriales	Lachnospiraceae	Lachnobacterium	
Firmicutes	Clostridia	Clostridiales	Ruminococcaceae	Ruminococcus	Ruminococcus gnavus
Firmicutes	Bacilli	Lactobacillales	Streptococcaceae	Lactococcus	
Firmicutes	Bacilli	Lactobacillales	Lactobacillaceae	Lactobacillus	
Firmicutes	Bacilli	Lactobacillales	Prcoccaceae	Streptococcus	
Firmicutes	Clostridia	Clostridiales	Unclassified Clostridiales		
Firmicutes	Bacilli	Lactobacillales	Enterococcaceae	Enterococcus	
Firmicutes	Clostridia	Eubacteriales	Peptostreptococcaceae	Clostridioides	C. difficile
Firmicutes	Negativicutes	Selenomonadales	Selenomonadaceae	Megamonas	
Firmicutes	Negativicutes	Acidaminococcales	Acidaminococcaceae	Phascolarctobacterium	Phascolarctobacterium faecium
Proteobacteria	Gammaproteobacteria	Pasteurellales	Pasteurellaceae	Haemophilus	
Proteobacteria	Gammaproteobacteria	Pseudomonadales	Moraxellaceae	Acinetobacter	
Proteobacteria	Deltaproteobacteria	Desulfovibrionales	Desulfovibrionaceae	Bilophila	
Proteobacteria	*Betaproteobacteria*	Burkholderiales	Sutterellaceae	Parasutterella	Parasutterella excrementihominis

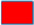
 taxa decreased with Vitamin D. 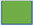 taxa increased with Vitamin D. 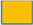 taxa that both increased and decreased with Vitamin D. 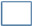 taxa not significantly altered with Vitamin D. Green represents the taxa that were increased with Vitamin D levels, and red represents the taxa that were decreased with Vitamin D levels. Orange represents the taxa that were both increased and decreased and white represents the taxa that were unchanged.

## Data Availability

Not applicable.

## Appendix A

**Table A1:** Search strategy for PubMed/SCOPUS/WOS/MEDLINE/OVID.

No.	Search Strategy
#1	Vitamin D AND microbiome
#2	Vitamin D AND microbiome AND immun *
#3	Vitamin D AND microbiome AND infection
#4	Vitamin D AND microbiome AND inflammat *
#5	Vitamin D AND microbiome AND profiling
#6	Vitamin D AND microbiome AND transcript *
#7	Vitamin D AND microbiome AND pediatric
#8	Vitamin D AND microbiome AND immune AND infection AND inflammation AND profiling AND transcript * AND pediatric

(*) Used as a truncation symbol

**Table A2:** Initial search strategy overview.

Databases	PubMed, SCOPUS, WOS, MEDLINE/OVID
Date of search	from January 1996 to January 2023
Study type	Books and documents were excluded
Limit	Conference abstracts were excluded
Other databases	Google Scholar was used with the same keywords as the main databases
